# Humoral and cellular immunity in response to an *in silico*-designed multi-epitope recombinant protein of *Theileria annulata*

**DOI:** 10.3389/fimmu.2024.1400308

**Published:** 2024-08-21

**Authors:** Asadullah Abid, Ambreen Khalid, Muhammad Suleman, Haroon Akbar, Mian Abdul Hafeez, Jawaria Ali Khan, Muhammad Imran Rashid

**Affiliations:** ^1^ Department of Parasitology, University of Veterinary and Animal Sciences, Lahore, Pakistan; ^2^ Institute of Microbiology, University of Veterinary and Animal Sciences, Lahore, Pakistan; ^3^ Department of Clinical Medicine and Surgery, University of Veterinary and Animal Sciences, Lahore, Pakistan

**Keywords:** immunoinformatics, multi-epitope, *Theileria annulata*, molecular docking, epitope prediction, flow cytometry

## Abstract

Tropical theileriosis is a lymphoproliferative disease caused by *Theileria annulata* and is transmitted by Ixodid ticks of the genus *Hyalomma*. It causes significant losses in livestock, especially in exotic cattle. The existing methods for controlling it, chemotherapeutic agents and a vaccine based on an attenuated schizont stage parasite, have several limitations. A promising solution to control this disease is the use of molecular vaccines based on potential immunogenic proteins of *T. annulata*. For this purpose, we selected five antigenic sequences of *T. annulata*, i.e. SPAG-1, Tams, TaSP, spm2, and Ta9. These were subjected to epitope prediction for cytotoxic T lymphocytes, B-cells, and helper T lymphocytes. CTL and B-cell epitopes with a higher score whereas those of HTL with a lower score, were selected for the construct. A single protein was constructed using specific linkers and evaluated for high antigenicity and low allergenicity. The construct was acidic, hydrophobic, and thermostable in nature. Secondary and tertiary structures of this construct were drawn using the PSIPRED and RaptorX servers, respectively. A Ramachandran plot showed a high percentage of residues in this construct in favorable, allowed, and general regions. Molecular docking studies suggested that the complex was stable and our construct could potentially be a good candidate for immunization trials. Furthermore, we successfully cloned it into the pET-28a plasmid and transformed it into the BL21 strain. A restriction analysis was performed to confirm the transformation of our plasmid. After expression and purification, recombinant protein of 49 kDa was confirmed by western blotting. An ELISA detected increased specific antibody levels in the sera of the immunized animals compared with the control group, and flow cytometric analysis showed a stronger cell-mediated immune response. We believe our multi-epitope recombinant protein has the potential for the large-scale application for disease prevention globally in the bovine population. This study will act as a model for similar parasitic challenges.

## 1 Introduction

Tropical theileriosis, caused by the intracellular protozoan parasite *Theileria annulata*, is an important tick-borne lymphoproliferative disease of cattle in tropical and subtropical regions of the world (1). It significantly affects the productivity of cattle. The disease spreads through the bite of *Hyalomma* ticks and is prevalent from the Mediterranean to the Middle East and parts of Asia (2). *T. annulata* is transmitted by *Hyalomma* ticks following cyclical development in the tick vector (3). In a vertebrate host (cattle), sporozoites enter lymphocytes, where they undergo transformation into the multinucleated schizont stage. From these schizonts, merozoites are subsequently released and invade circulating erythrocytes, developing into the piroplasm stage (4). Theileriosis is a major concern for the dairy industry as it severely affects the productivity of exotic cattle, thus causing significant economic loss to the livestock sector due to high morbidity and mortality (3, 5). The disease is prevalent among all age groups of cattle but it is more pronounced in older animals (3) and the usual signs include pyrexia, anemia, and severe enlargement of the superficial lymph nodes (6). During microscopic examination, this parasite appears to be a round, oval rod-like, or irregularly shaped organism in lymphocytes and erythrocytes (7).

Current strategies for controlling tropical theileriosis include the use of acaricides for vector control, chemotherapeutic agents against the parasite, such as buparvaquone, to target the early infection stages (8), and vaccination against vectors to block its transmission to the host or against the parasite (9). The indiscriminate use of chemotherapeutic agents, such as buparvaquone, against the parasite has resulted in the emergence of resistant parasitic strains (10). Hence, disease control through vaccination is inevitable (11). Two main strategies are being used to vaccinate the cattle against *Theileria* infection: live attenuated parasites and antigen-based vaccines (12). Vaccination with attenuated schizont of *T. annulata* is considered a promising measure for controlling tropical theileriosis; this vaccine provides partial immunity against heterologous strains under experimental conditions. The vaccinated animals, despite surviving, may suffer clinical symptoms of acute tropical theileriosis (13). The attenuated cell line vaccine has conferred variable degrees of protection in different trials. In one study, calves withstood the parasitic challenge for up to 6 months (14), whereas in another trial, four calves out of seven died of a challenge infection 90 days during or after immunization (15). The subunit vaccines are believed to provide variable degrees of protection (12). The TaSP protein alone does not confer protection but it provides a synergistic effect to the attenuated Atbara cell line vaccine (3), and SPAG-1 confers only partial protection to sporozoites-based challenge infection (16).

The mechanism of protection against *T. annulata* is mainly cell-mediated and particularly directed against the schizont stage (3). Although CD4+ and CD8+ T-cells are important in protection, cytotoxic (CD8+) T-cells are not induced effectively in *Theileria* infection (17). Immunization with live parasites does not prevent infection completely as schizont-infected cells are detectable 10 days post-challenge in vaccinated calves (13).

Given the practical challenges, such as maintenance of the cold chain, reversion to the pathogenic form, and the occurrence of disease in immunocompromised individuals, the live vaccine has drawn less attention (13), whereas the subunit vaccines are believed to be safer and more economical (18) and carry the potential to fill this gap. Some macroschizont stage live attenuated cell line vaccines are believed to provide solid immunity against tropical theileriosis but their manufacturing time and cost for attenuations are principal limiting factors (19). Although some toxicity issues can arise, molecular vaccines are regarded as safer, economical, and identical from batch to batch with zero risk of a reversion to virulence, over-attenuation or being carriers of contaminating pathogens of culture origin. A number of potential vaccine candidates have been studied for inducing the protective immune response against *T. annulata*. Tams produced in *Escherichia coli* was recognizable by *T. annulata*-immune calf serum, and immunization with this antigen also reduced the severity of clinical signs in experimental animals (20). SPAG-1 elicited partial protection in calves (16), and TaSP antigen improved the efficacy of attenuated cell line vaccine (3). Now, the focus of vaccination is on defined parasitic antigens of sporozoites and intracellular schizont stages of the parasite (13) as the small antigenic sequences of a protein can also trigger an immune system (21), but the effectiveness of these vaccines needs to be improved. Although experimental methods used for characterizing epitopes are time-consuming and require huge resources, the successful outcome in terms of protection against the disease outweighs all these challenges.

The availability of epitope prediction methods can rapidly aid researchers in simplifying this problem (22). Immuno-bioinformatics (immune-informatics) has served as an aid in the targeting of potential molecules and has scaled down a wide range of potential molecules for vaccine purposes; thus, it helps in finding superior antigenic candidates with a higher probability of becoming effective vaccines (23). Our study focused on developing a peptide-based multi-epitope construct against *T. annulata* using immunoinformatic approaches.

In the present study, we formulated a chimeric construct using epitopes from five immunogenic sequences, SPAG-1, TaSP, Tams, Spm2, and Ta9 of *T. annulata*, for vaccine development. We hypothesized that our vaccine based on this chimeric construct induces protective humoral and cellular immunity against different stages of *T. annulata*.

## 2 Materials and methods

### 2.1 Sequence retrieval, antigenicity score assessment, and epitope prediction

The amino acid sequences of five different immunogenic proteins were retrieved from the NCBI database in FASTA format (accession numbers are shown in **Table 1**). CLUSTALW alignment of protein sequences and consensus sequences were obtained via DNASTAR Lasergene software and SCRATCH Protein Predictor (http://scratch.proteomics.ics.uci.edu/) was used to screen the antigenic potential of these candidate proteins (24). Cytotoxic T-lymphocyte (CTL) epitopes of these proteins were predicted by the NetCTL 1.2 server (http://www.cbs.dtu.dk/services/NetCTL/); A2, A3, and B7 allelic supertypes were selected and had 88.3% total population coverage worldwide (25). A threshold of 0.75 was set (26), with default settings in other parameters. These predicted epitopes were subjected to the Immune Epitope Database (IEDB) (http://tools.iedb.org/immunogenicity/) (27) to check their immunogenicity. The CTL epitopes with higher positive scores were considered for multi-epitope construct design. The ABCPred server (https://webs.iiitd.edu.in/raghava/abcpred/ABC_submission.html) was used to predict linear B-cell epitopes. A threshold of 0.5 was chosen with default settings. Epitopes were predicted based on their scores from 0 to 1. The higher the score, the higher the probability of being an epitope. The IEDB server (http://tools.iedb.org/mhcii/) was used to predict the helper T-lymphocyte (HTL) epitopes (15-mers) of candidate proteins using the MHC class II module. These epitopes were predicted with a reference set of HLA alleles. This prediction was based on IC50 values and percentile rank/adjusted rank. The peptide with a lower percentile rank has a high affinity against HTL epitopes.

**Table 1:** The retrieved amino acid sequences from the NCBI database, with accession numbers.

Antigens	Accession numbers
**SPAG-1**	ADK09902.1, AAA30134.1
**spm2**	CAA75787.1, XP_952546.1, CAI74814.1, QCE32093
**Ta9**	ADX60654.1, ADX60653.1, ADX60652.1, ADX60651.1, ADX60650.1, ADX60649.1, ADX60648.1, ADX60647.1, ADX60646.1, ADX60645.1, ADX60644.1, ADX60643.1
**Tams 1**	AYA72340.1, AYA72339.1, AHB14328.1, AHB14327.1, QTG10693.1, ANW07459.1, ANW07455.1, ANT82210.1, ANT82209.1, ANT82208.1, ANT82207.1, AZJ17473.1
**TaSP**	XP_952743.1, CAI76117.1, AEX58667.1, ABS88736.1, ABS88735.1, ABS88734.1, ABS88733.1, ABS88732.1, ABS88731.1, ABS88730.1, ABS88729.1, ABS88728.1, ABS88727.1, ABS88726.1, ABS88725.1, ABS88724.1, ABS88723.1, ABS88722.1, ABS88721.1, ABS88720.1, ABS88719.1, ABS88718.1, ABS88717.1, ABS88716.1, ABS88715.1, ABS88714.1, ABS88713.1, ABS88712.1, ABS88711.1, ABS88710.1

### 2.2 Construction of a chimeric single multi-epitope sequence

The predicted epitopes were fused together with suitable linkers reported previously (28, 29) for the chimeric multi-epitope construct. GPGPG, AAY, and KK linkers were used to link HTL, CTL, and B-cell epitopes, respectively. An adjuvant 50S ribosomal protein L7/L12 (accession number P9WHE3) retrieved from UniPort was attached toward the N-terminal end of the construct. EAAAK linker was used to join the adjuvant with the sequence. A 6xHistag was also incorporated at its C terminus.

### 2.3 Allergenicity and antigenicity determination

AllerTop v2.0 (https://www.ddg-pharmfac.net/AllerTOP/method.html) was used to determine the allergenicity of the multi-epitope construct. The Vaxijen server (http://www.ddg-pharmfac.net/vaxijen/) was used to evaluate the antigenicity of the multi-epitope construct. For parasitic models, a threshold of 0.5 was used to predict the protective antigens (30, 31). There is organism-to-organism variation, and the accuracy of this server ranges from 70% to 89%.

### 2.4 Evaluation of physicochemical properties

The ProtParam tool of ExPASy (https://web.expasy.org/protparam/) was used for the physical and chemical properties of the multi-epitope construct. Its computation is based on the protein sequence and pKa values of amino acids in the protein sequence (32). This tool allows the evaluation of the amino acid composition, molecular weight, theoretical isoelectric point (pI), instability index, aliphatic index, and grand average of hydropathicity (GRAVY).

### 2.5 Prediction of secondary and tertiary structure

The PSIPRED server was used to predict the secondary structure of the multi-epitope construct (33). The amino acid sequence was provided as an input sequence. PSI BLAST (http://bioinf.cs.ucl.ac.uk/psipred/) was performed to determine the relative sequences and homology. The secondary structure consists of an α-helices, beta strands, and a coil. The tertiary structure of the designed construct was predicted by the RaptorX (http://raptorx.uchicago.edu/ContactMap/) structure prediction server (34). The FASTA format sequence of the construct design was used as an input to generate a tertiary structure.

### 2.6 Refinement and validation of the three-dimensional structure

The 3D-refine web server (https://sysbio.rnet.missouri.edu/3Drefine/) was used to refine the three-dimensional structure of the multi-epitope construct. Moreover, a Ramchandran plot was created using RAMPAGE (http://mordred.bioc.cam.ac.uk/~rapper/rampage.php) so that the tertiary structure could be validated.

### 2.7 Molecular docking study of our construct and receptor

To predict the binding energy of our multi-epitope construct with the receptor, a molecular docking study was carried out between TLR2 (PDB ID: 5D3I) as a receptor and the refined tertiary structure of the multi-epitope construct of *T. annulata* as a ligand. The ClusPro2.0 server (https://cluspro.bu.edu/publications.php) was used to generate the outcomes of a construct-receptor complex (35–38). This server used the PDB files of the receptor and the ligand; a fast Fourier transform algorithm provided the models with electrostatic energies. After molecular docking, a docked molecular complex with the least binding energy was selected for further analysis.

### 2.8 Codon optimization and *in silico* cloning of the designed construct

**Figure 1:**
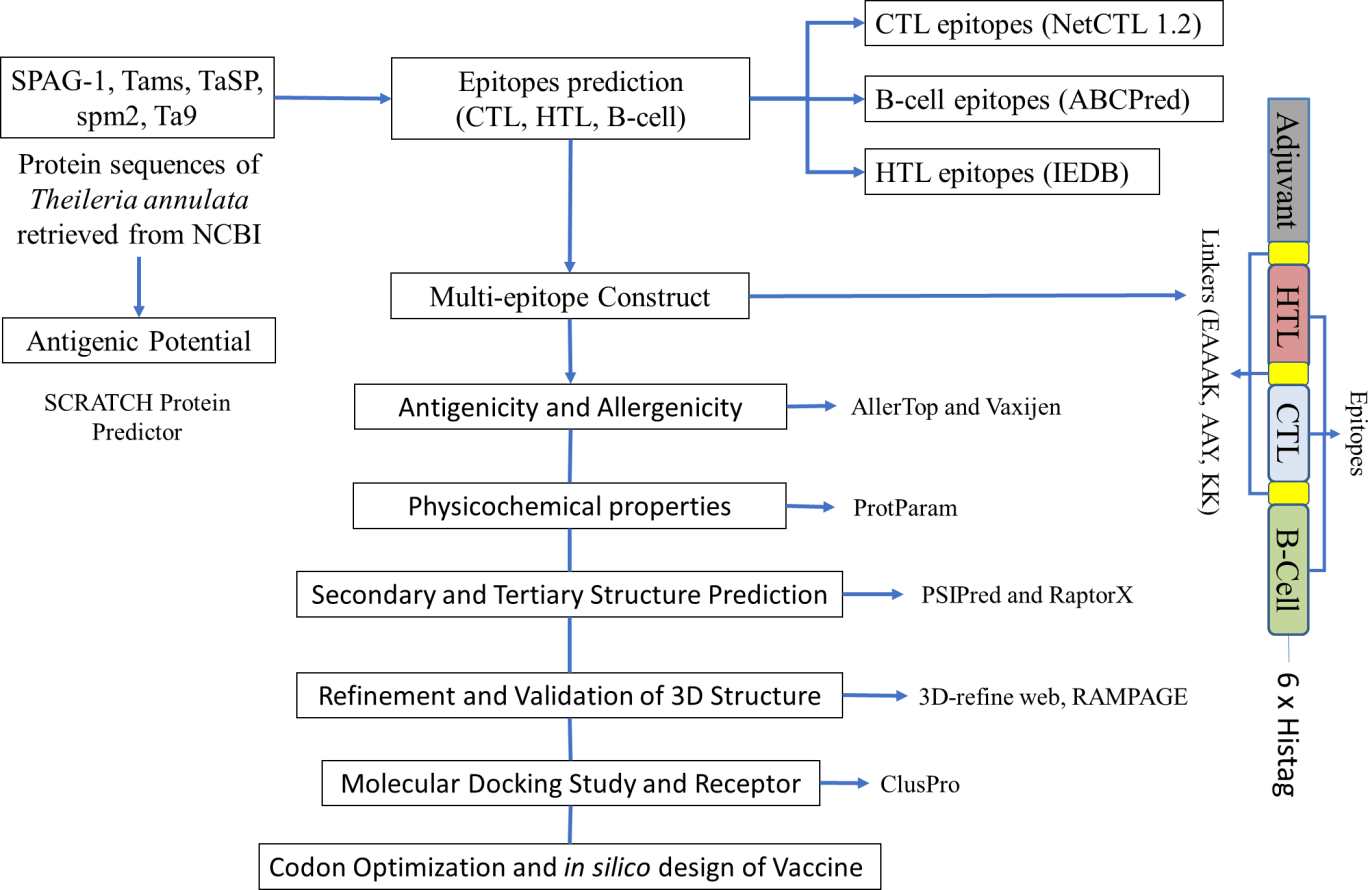
A schematic diagram of the multi-epitope immunogenic construct and tools used for the immunoinformatic analysis of *Theileria annulata*.

The Java Codon Adaptation Tool (JCAT) (http://www.jcat.de/) was used to determine the expressibility of our designed construct in *E. coli* (strain K12). JCAT provides an improved nucleotide sequence that will have a higher probability of expression in *E. coli* (strain K12). The construct sequence was used as an input and the *E. coli* K12 strain was chosen as a host for expression. While running the JCAT tool, we selected additional options to avoid rho-independent transcription terminators, prokaryotic ribosome-binding sites, and cleavage sites for our restriction enzymes (39, 40). The reverse transcription was carried out using the Sequence Manipulation Suite (https://www.bioinformatics.org/sms2/rev_trans.html). After the reverse transcription, we used the JCAT tool to optimize the codon and determine the codon adaptation index (CAI) and GC content. The JCAT output was analyzed for the presence of commercially available restriction enzymes with the help of NEBcutter (http://nc2.neb.com/NEBcutter2/). Open reading frames were also checked using the Sequence Manipulation Suite (https://www.bioinformatics.org/sms2/orf_find.html). *In silico* cloning was performed using SnapGene, and the pET28a plasmid had the optimized sequence cloned into it. pET-28a-*T. annulata* was ordered from Genscript (Piscataway, USA). **Figure 1** shows the design of our multi-epitope construct.

### 2.9 Expression and characterization of the synthetic DNA transformation

#### 2.9.1 Competent cells preparation and the transformation of the plasmid

We prepared 4% Luria–Bertani (LB) agar and 2% LB broth in distilled water and autoclaved them. Under sterile conditions, mother culture was prepared by streaking BL21 (DE3) cells (Thermo Fisher, cat. no. C600003) on an agar plate. The plate was incubated at 37°C for 24–48 h and growth was observed after 24 h. Subsequently, a subculture was prepared under the same conditions from a single colony of mother culture. A primary culture was prepared by mixing a single colony from a subculture plate with liquid broth, which was then placed in a shaker incubator (Robus Technologies, Cat. UV1100) at 37°C, 0.66 × g, until the OD_600_ value reached 0.6. Secondary culture was prepared by adding 50 µl of primary culture in 200 ml of LB broth in the same conditions until the OD_600_ reached 0.6. Aliquots of 10 ml were centrifuged at 7,000 g at 4°C for 5 min, after which, the supernatant was discarded and the pellets were washed three times and stored in 50 µl of 1.5% glycerol at −20°C.

Two aliquots were thawed and centrifuged, and supernatant glycerol was removed and the pallet was mixed with 50 µl of chilled distilled water separately. We added 2 µl (100 ng) of plasmid in one vial, and the second vial without plasmid was used as a negative control. Both samples were transferred into 0.1 mm cuvettes separately and the plasmid was transferred by electroporation using a gene pulser (Bio-Red Gene Pulser Xcell Electroporator System) at 1.8 kV, 25 µF, and 200 Ω. Then, 800 µl of super optimal medium with catabolic repressor was added in each cuvette and the samples were transferred to a microcentrifuge tube and kept in a shaker incubator for 40–60 min at 30°C. The samples (300 µl each) were spread on LB agar plates that contained kanamycin (50 µg/µl) as a selection marker. The plates were kept in an incubator at 37°C for 24-48 h and growth was observed after 24 h.

#### 2.9.2 Restriction analysis

A single colony from the positive control LB plate was added to 50 ml of LB broth and kept in an incubator at 37°C with shaking at 220 rpm until its OD_600_ value reached 2.0. The culture was then placed in a 50 ml Falcon tube and plasmid extraction was carried out using a GeneJet Plasmid Miniprep kit (K0503) according to the manufacturer’s instructions to confirm the transformation of the *T. annulata* sequence in our plasmid by restriction analysis. The purified plasmid was stored in a 1.5 ml tube at −20°C. The plasmid was verified on a 0.9% agarose gel. Plasmid digestion was carried out using the restriction enzymes NdeI and XhoI to check the presence of cloned genes. A 10 µl volume of plasmid was mixed in 1 µl of NdeI, 0.5 µl of XhoI, and 1 µl of B4 buffer. Bovine serum albumin (BSA) was added to increase enzyme activity. The sample was placed in a water bath at 37°C for 3 h. After digestion, the restriction sites were analyzed on a 0.9% Agarose gel.

#### 2.9.3 Expression and induction

A single colony of transformed plasmid culture (pET28a-*T. annulata*) was taken and added to 60 ml of LB broth and placed in shaker incubator at 37°C and 0.66 × g until the OD_600_ value reached 0.6. Then, 14.29 mg (1mM) of isopropyl β-D-1-thiogalactopyranoside (Cat. No. 367-93-1) was added to 60 ml of culture and kept in shaker incubator for 4 to 24 h at 37°C and 0.66 × g. A 2 ml volume of culture was collected and stored in 2 ml tubes every 2 h and stored at 4°C.

#### 2.9.4 SDS-PAGE and western blotting

SDS-PAGE was performed on a 12% resolving gel to separate purified, induced, and BL21 without plasmid proteins in different wells. The sample was mixed in equal volumes of 2× Laemmli buffer in separate tubes and boiled for 10 min prior to electrophoresis. Two gels were prepared and 5 µl of protein marker (Cat# 26616) was added to estimate the size of our protein. Gel was stained with Coomassie Blue for 1 h. Excessive stain was destained overnight with destaining solution and protein bands were observed. The other gel was used for the development of the blot on nitrocellulose membrane (NCM) (LC2001, Invitrogen, Germany) to confirm the specific *T. annulata* proteins using serum of an animal that had recovered from theileriosis as a source of primary antibodies at 1:1000 dilutions in skimmed milk. The NCM was treated with anti-ovine secondary antibodies at 1:30000 dilutions in 5% skimmed milk. An aliquot of 5-bromo-4-chloro-3-indolyl phosphate/p-iodonitrotetrazolium was added as a substrate for 15 min. The reaction was stopped with excessive water.

### 2.10 Protein quantification

The quantification of proteins was carried out using a bicinchoninic acid (BCA) kit (Cat. 20831001) as per the manufacturer’s instructions. The working principle of this method is that in alkaline solution proteins reduce Cu^+2^ to Cu^+1^ and as a result purple color forms due to BCA.

### 2.11 Immunization in an animal model

Two rabbits and twelve calves were used in the present study. First, one rabbit was inoculated intramuscularly with 10 µg of recombinant protein of *T. annulata* following the methodology of Hagiwara et al. (41). Another dose (10 µg) was injected 3 weeks apart. The other rabbit was kept as a control. The sera of both rabbits were collected 3 weeks after the second dose to check the antibody titers through an ELISA. The calves were divided into three groups; the first group consisted of healthy animals as a negative control (n=3), the second group consisted of immunized animals (n=3), and the third group [infected control (n=6)] comprised three naturally infected calves and three experimentally infected calves. Recombinant muti-epitope *T. annulata* protein (100 µg) was injected subcutaneously into the immunized group. The sera for the ELISA and whole blood for flow cytometry were collected 3 weeks after immunization. The animals were reared in an experimental station at the University of Veterinary and Animal Sciences (UVAS), Lahore, under natural climatic conditions and received *ad libitum* feed and water.

### 2.12 ELISA

The recombinant protein (0.125 µg/ml) of multi-epitope of *T. annulata* was coated on a 96-well plate (JET BioFil, China) in 50mM of Na_2_CO_3_ followed by overnight incubation (4°C). The plate was washed five times with 0.05% Tween 20 in 1× PBS buffer (300 µl per well). The blocking was carried out with 4% BSA in 1× PBS (200 µl per well) followed by incubation (37°C for 2h.) and washing (five times) with washing buffer (42). Two wells of the plate were used as a negative control, two wells for a positive control, and another two wells were kept blank. The plate was incubated followed by washing. Alkaline phosphatase (AP)-anti-bovine IgG (whole molecule) antibodies produced in rabbits (Sigma-Aldrich, Saint Louis, MO, USA, Cat # A0705) were added (1:10,000 dilution, 100 µl per well) and incubated. After washing, p-nitrophenyl phosphate (Thermo Scientific, USA) was added (100 µl per well), followed by incubation. Then, 1M NaOH (100 µl per well) was added to stop the reaction and OD_450_ was measured using an ELISA reader (ELX-800, BioTek, USA).

### 2.13 Flow cytometry

Blood was collected from the jugular veins of calves from the immunized group (n=3, and processed individually for flow cytometric analysis as described previously (43) at day-0 and day-21 post-immunization. Briefly, 10 ml of blood was collected and centrifuged at 2,400 g for 20 min. The buffy coat was separated carefully with a small volume of red blood cell (RBC) layer. The sample was treated with RBC lysis buffer for 10 min and centrifuged again. The pellet was resuspended in PBS, and the viability of the cells was above 95%, as determined by Trypan Blue. An aliquot of 0.5 million cells was blocked with 4% fetal bovine serum and stained with antibodies (in 1% BSA prepared in PBS) according to the manufacturer’s instructions. Anti-bovine monoclonal anti-CD4 antibodies (Cat. MA1-80176; Thermo Fisher Scientific, USA) were used for the detection of CD4+ T-cells, and monoclonal anti-CD8-antibodies (Cat. MA1-80900; Thermo Fisher Scientific, USA) were used to detect bovine CD8+ T-cells. Non-specific isotype controls were used: a mouse IgG2a, PE (Cat. PA5-33207; Invitrogen, USA) and mouse IgG2a, FITC (Cat. PA5-33239; Invitrogen, USA) were used at the same concentration as the primary antibodies. After staining, the cells were washed three times and preserved in 2% paraformaldehyde (Sigma-Aldrich, St. Louis, MO, USA), before processing by flow cytometry. An Attune NxT Acoustic Focusing Cytometer (Invitrogen^®^, Waltham, MA, USA) was used for sample acquisition; 10,000 events per sample were recorded and analyzed with NxT software (version 2.7). An Attune NxT Flow Cytometer was used to quantify CD4+ and CD8+ lymphocytes pre- and post-vaccination.

### 2.14 Statistical analysis

The difference in antibody titers was analyzed using the Mann–Whitney test, and a paired Student’s t-test was used to compare T-cells. Statistical differences were considered significant at p ≤ 0.05 a *priori*. Data were analyzed using GraphPad Prism 7 for Mac OS X (GraphPad Software, La Jolla, CA, USA, www.graphpad.com).

## 3 Results

We selected five immunogenic proteins of *T. annulata* that are released from time to time during the development of the parasite in the host and can elicit humoral and cell-mediated immune responses. Therefore, the desired immunity can be achieved against all stages of this parasite. SPAG-1 is an immunodominant protein that results in delayed clinical symptoms and attenuates the virulent challenge (17). Tams is a merozoite surface antigen and provides protection in small-scale vaccination trials (3). TaSP is an immunodominant protein expressed in the sporozoite and schizont stages (44). Spm2 is expressed in the sporozoite, macroschizont-infected leucocytes, and piroplasm stages (45). Ta9 is released during macroschizont development and detected by humoral and cell-mediated responses (46, 47).

### 3.1 Sequence retrieval, antigenicity score assessment and epitope prediction

The amino acid sequences of SPAG-1, Ta9, Tams-1, spm-2, and TaSP of *T. annulata* were retrieved from the NCBI database and selection was based on individual antigenic scores using the SCRATCH Protein Predictor. CTL epitopes from supertypes A2, A3, and B7 with a combined score >0.75 were predicted and selected from the NetCTL 1.2 server. Seven CTL immunogenic epitopes (9 mers) were identified by the IEDB. Epitopes with the highest immunogenicity scores were selected for the construct design. Seven B-cell epitopes (score ≥0.90) were selected for the design of the multi-epitope construct using the ABCPred server. MHC II-specific HTL epitopes were predicted by the IEDB server for the HLA allele, which has 99.9% population coverage worldwide. Six HTL epitopes (15-mer length) with the lowest percentile ranks were selected for the construct design. **Table 2** summarizes the predicted scores and epitopes.

**Table 2:** Summary of the predicted antigenic score, selected epitopes of CTL, B-cell and HTL epitopes.

Antigen	Antigenic score	CTL epitopes		Linear B-cell epitopes		HTL epitopes	
		Epitope	Score	Sequence	Score	MHC II-binding peptide	PR ^4^/AR ^5^
**SPAG-1**	0.934172	SLVQTLINL^1^	1.1125	SGPIPSPGDPRAITGQ	0.96	LHFLLTIPAIFVSGA	0.67
				SEIINKKGTEDQDQTS	0.94	MNILHFLLTIPAIFV	0.79
**Ta9**	0.864405	LLTPGIILY^2^	1.2540	DGDSDGDGDGDSMLPP	0.97	MNLLTPGIILYSFYL	6.00
				PGPPSGPPTEAPDGTK	0.94		
**Tams-1**	0.864052	RLKETYFEL^1^	1.0673	VVRLDYFYTGDSRLKE	0.90	AVFASVLIVFSSVLY	3.60
**Spm-2**	0.778585	LIPAFTAFV^1^	1.2342	KQTPDSTTSFRTRNNF	0.94	EFILRYATNLGSDVQ	1.80
		NPGENIGPL^3^	1.4240				
**TaSP**	0.714529	YLFVLFPIL^1^	1.3585	QEPIESPQQPTQPSTQ	0.95	HLFLFLIAFCAYALD	0.67
		FVLFPILLK^2^	1.6118				

^1^ Supertype A2; ^2^ Supertype A3; ^3^ Supertype B7; ^4^ Percentile Rank; ^5^Adjusted Rank.

### 3.2 Design of the multi-epitope construct

**Figure 2:**
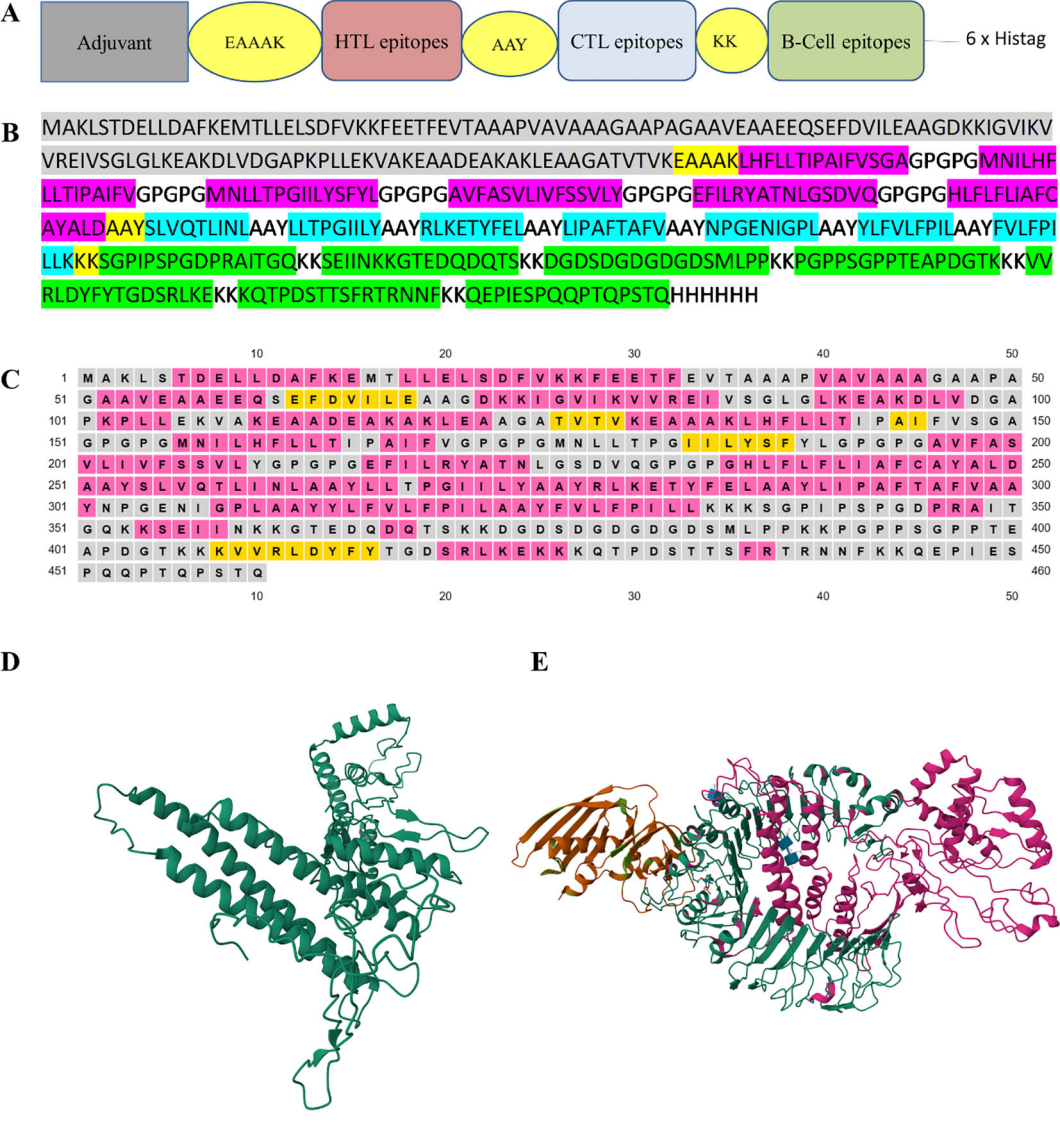
**(A)** The design of multi-epitope construct with EAAAK, AAY and KK linkers. **(B)** The construct consists of 460 amino acids in which the linkers help join the epitopes with the adjuvant and with themselves. B-cell epitopes are represented in green, CTL epitopes in blue, HTL epitopes in red, and the adjuvant is in gray. **(C)** The secondary structure map, in which protein structures are coded in colors for the α-helix (pink), β-strands (yellow), and coil (gray). **(D)** The refinement of the model using the 3D-refine web server. **(E)** The docked model was obtained using ClusPro (https://cluspro.bu.edu). The lowest energy value obtained from the ClusPro calculation was −1072.5. Our designed construct is in pink and its affinity with the TLR2 receptor is evident. The three-dimensional images were created using RCSB PDB – 3D View (https://www.rcbs.org/3d-view).

The final multi-epitope construct consisted of 460 amino acids that comprised an adjuvant and six HTL, seven CTL, and seven linear B-cell epitopes. Furthermore, there was one EAAAK linker, seven KK linkers, seven AAY linkers, and five GPGPG linkers, as shown in **Figures 2A, B**.

### 3.3 Antigenicity, allergenicity, physicochemical and structural properties, and molecular docking of the designed construct

The probability score of the chimeric construct for its putative antigenicity was 0.5518 during analysis with the VaxiJen v2.0 server, indicating it as a probable antigen. The construct was also verified on the AllerTop v2.0 server, revealing its non-allergenic nature. Both of these characteristics demonstrate the antigenicity and safe potential of the designed construct. The designed construct was composed of 460 amino acid residues and its molecular weight was 48.86 kDa with a theoretical pI of 5.42, suggesting its acidic nature. The construct consisted of 51 negatively charged and 45 positively charged amino acid residues. The instability index was computed to be 32.04 and this classified the protein as stable (<40). The aliphatic index of a protein determines the volume occupied by aliphatic side chains and its value was 94.07, demonstrating the thermostable nature of the construct. The GRAVY value of the construct was 0.062, showing the hydrophobic characteristic of the designed construct. Our designed model consisted of 51.30% α-helices, 6% β-strands, and 42.6% random coil elements, as determined by analysis with PSIPRED (**Figure 2C**), and the tertiary structure of the designed construct was analyzed using the RaptorX prediction server. The 3D-refine web server was used to refine the tertiary structure of the designed construct (**Figure 2D**). The Ramachandran plot validated the tertiary structure and showed the structure had 78.0%, 19.1%, and 1.9% residues in the most favorable region, allowed region, and general region, respectively, and only 1.1% in the disallowed region. The best docked model with the lowest energy score was chosen using ClusPro (**Figure 2E**).

### 3.4 In silico cloning of the final multi-epitope construct into an expression vector

We first performed codon optimization before cloning the designed construct into a suitable vector using the *in silico* method. The CAI and GC content of the improved sequence were 0.951 and 52.53%, indicating a higher probability of expression. The restriction enzymes NdeI and XhoI were chosen and were located in the multiple cloning site of the plasmid vector pET28a. Finally, *in silico* cloning was performed with the help of the SnapGene tool.

### 3.5 Restriction analysis and production of recombinant proteins

**Figure 3:**
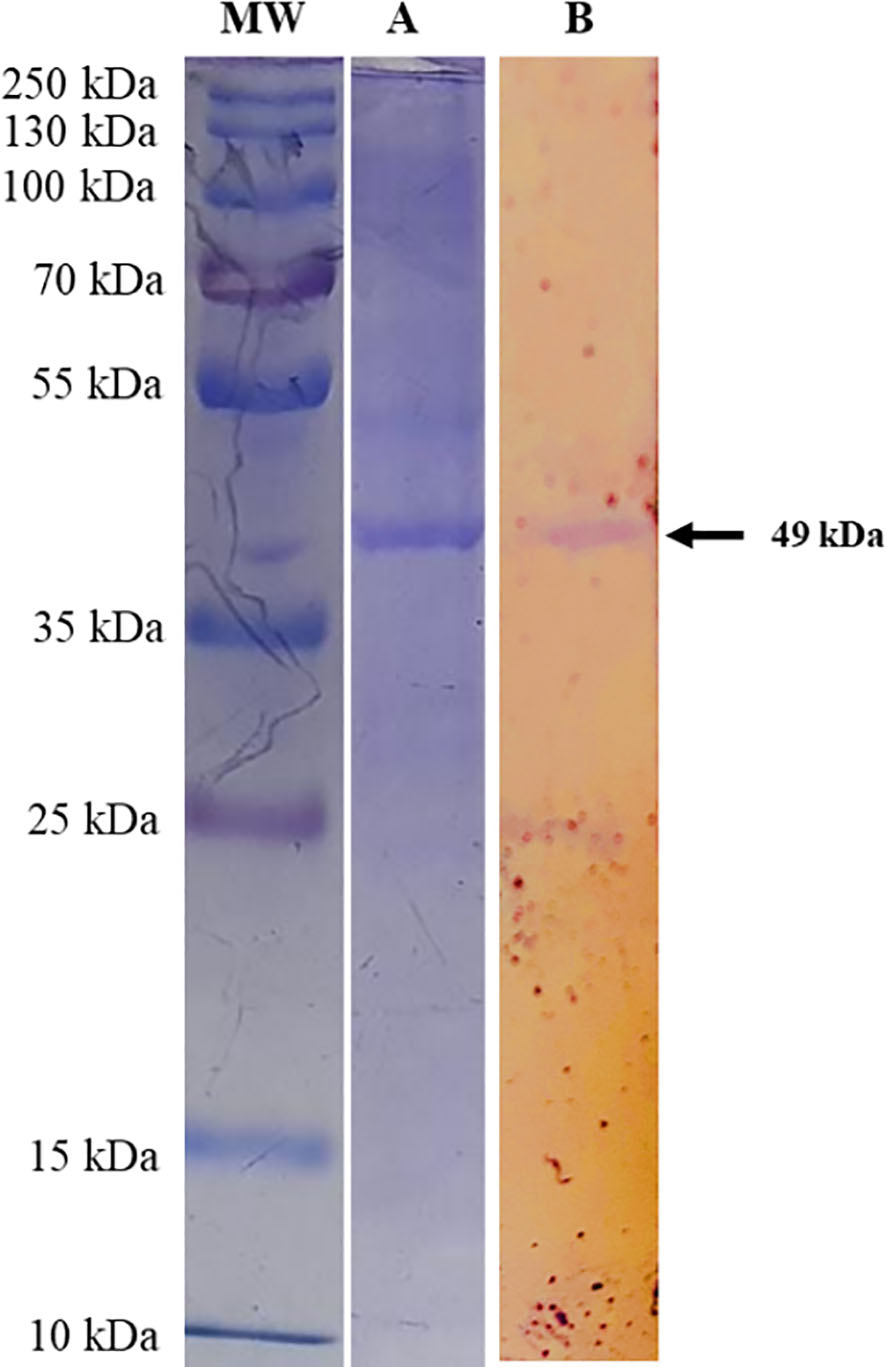
SDS-PAGE analysis of *Theileria annulata*-multi-epitope-protein expressed from the designed construct transformed into BL21, using a 12% acrylamide gel. Lane MW shows a protein marker (cat # 26619) and Lane **(A)** shows the multi-epitope protein from the transformed BL21. Lane **(B)** shows the western blot analysis, which demonstrates the reactivity of *Theileria annulata*-purified-multi-epitope-protein using sera from cattle (recovered from theileriosis) and developed using anti-bovine IgG antibodies-AP (cat # A0705, Lot # 113M4819V).

The multi-epitope sequence of *T. annulata* was analyzed for restriction sites using NEB cutter and no restriction sites were found for Nde1 and Xho1. Therefore, an Xho1 site was incorporated into the 5′ end of the forward primer and an Nde1 site was incorporated into the 3′ end of the reverse primer. The BL21 was transformed with pET28a-*T. annulata* and the transformed colonies were confirmed on LB agar supplemented with kanamycin. After expression and purification, the recombinant protein of multi-epitope *T. annulata* was subjected to SDS-PAGE using a 12% gel. A band of 49 kDa was observed after Coomassie Blue staining (**Figure 3A**). The expressed and purified recombinant protein was also reacted with sera of theileriosis in western blotting, and a band of 49 kDa can be observed in **Figure 3B**.

### 3.6 Antibody titers of rabbit and calf sera

**Figure 4:**
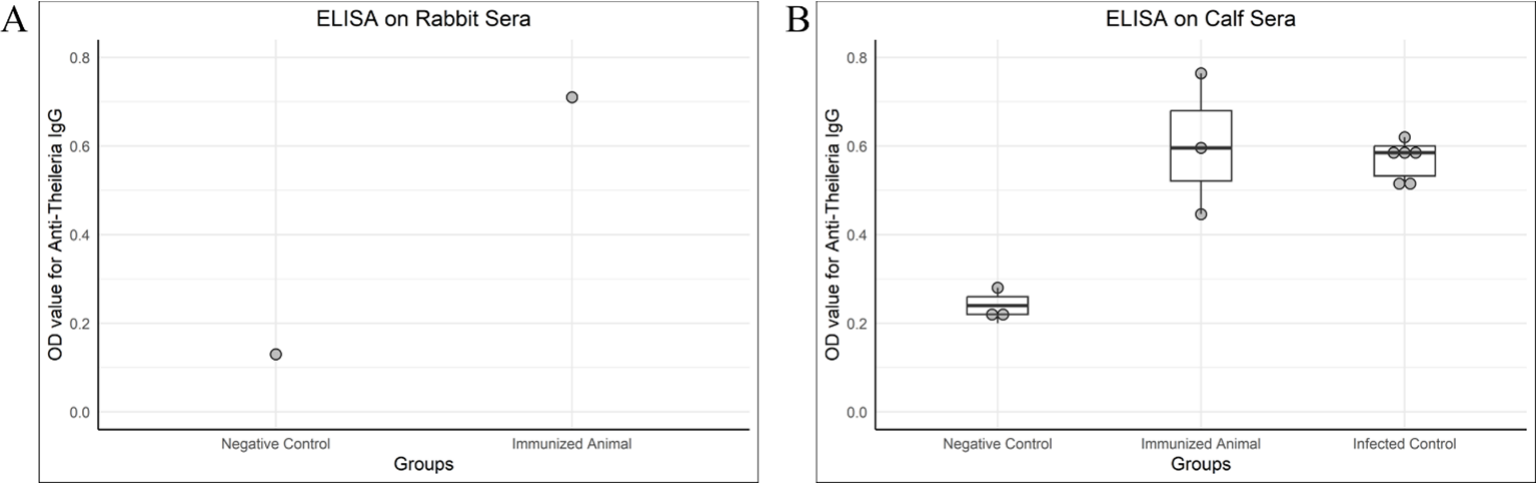
OD values of antibody detection ELISA (IgG) on rabbit and calf sera. **(A)** A rabbit was given two doses of chimeric antigen (10 µg/dose) 3 weeks apart, intramuscularly. The serum for IgG titer was collected 3 weeks after the second dose. A negative control serum was collected from a healthy rabbit negative for *Theileria* on PCR. The immunized rabbit was also healthy and negative for *Theileria* by PCR but given two doses of chimeric antigen. **(B)** Negative control and immunized calves were healthy and negative for *Theileria* in PCR, whereas the infected control was a naturally infected calf. The calves administered with 100 µg of chimeric protein were named as an immunized group, the sera of which was tested 3 weeks post-immunization for IgG. The serum of the immunized calf was collected 3 weeks post-immunization.

The antibody titer against *T. annulata* was high in immunized rabbit and calves compared with control animals, as shown in **Figure 4**.

### 3.7 Flow cytometric analysis

**Figure 5:**
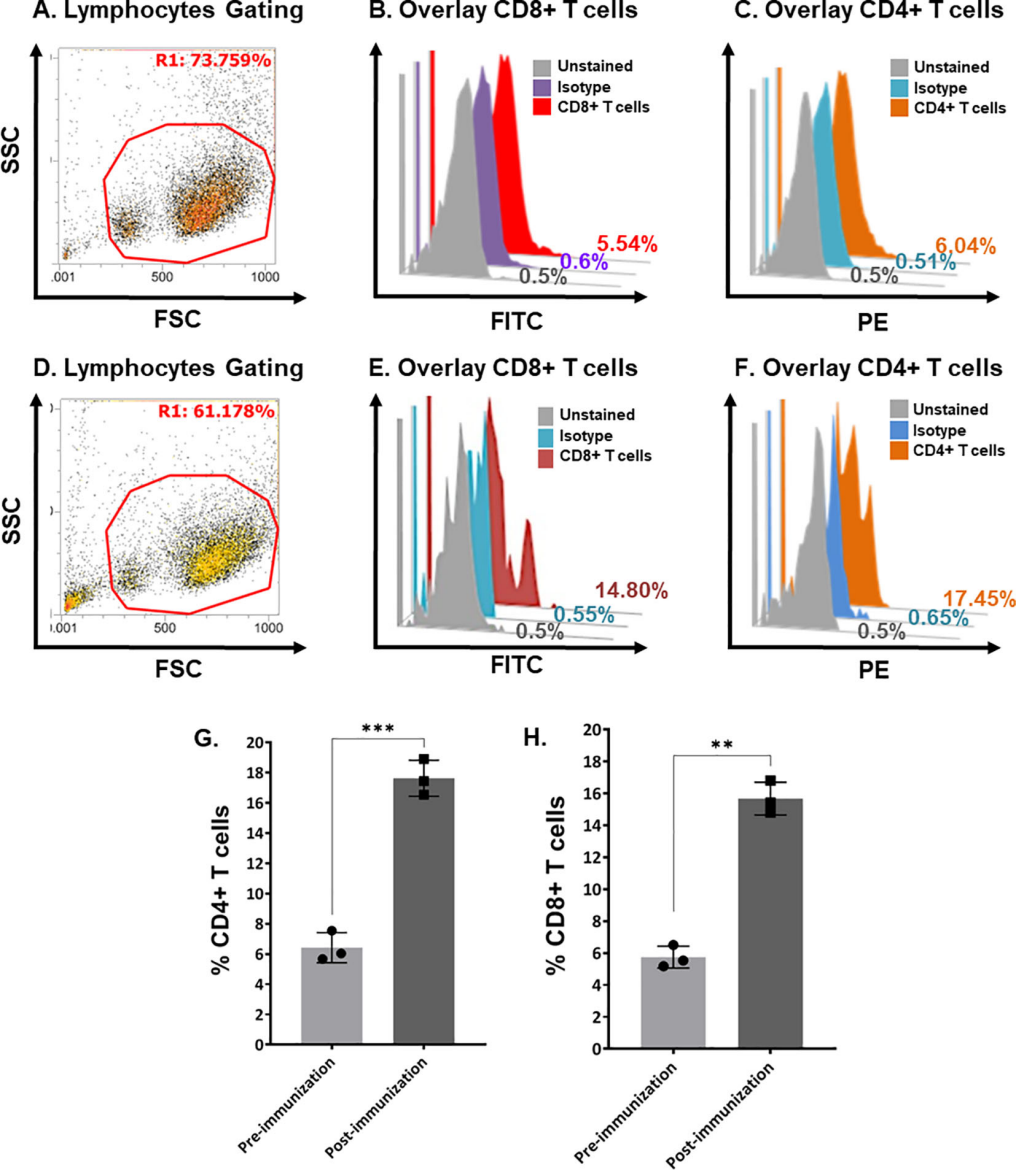
Quantification of bovine CD4+ and CD8+ T-cells in pre- and post-immunization calves using flow cytometry. **(A)** Scatter plot showing the gating of lymphocytes in bovine buffy coat cells in pre-immunized calves. **(B)** Histogram overlay showing unstained cells (gray), isotype control (purple), and CD8+ T-cells (Red) in pre-immunized calves. **(C)** Histogram overlay showing unstained cells (gray), isotype control (blue), and CD4+ T-cells (brown) in pre-immunized calves. **(D)** Scatter plot showing the gating of Bovine buffy coat cells in post-immunized calves. **(E)** Histogram overlay showing unstained cells (gray), isotype control (blue), and CD8+ T-cells (maroon) in post-immunized calves. **(F)** Histogram overlay showing cells unstained cells (gray), isotype control (blue), and CD4+ T-cells (brown) in post-immunized calves. **(G)** Bar graph shows a significant increase in CD4+ T-cells at day-21 post-immunization compared with day-0 in calves (p < 0.0005). **(H)** Bar graph shows a significant increase in CD8+ T-cells at day-21 post-immunization compared with day-0 calves (p < 0.005). FSC, forward scatter; SSC, side scatter; PE, phycoerythrin; FITC, fluorescein isothiocyanate. The stars show the level of significance '***' (p < 0.0005) and '**' (p < 0.005). The lesser the p value, the more significant results are.

Flow cytometric analysis quantified the cell-mediated immune response. Helper T-cells and cytotoxic T-cells were increased. **Figure 5** shows the quantification of CD4+ and CD8+ T-cells in pre-immunized calves (**Figures 5A–C**) and post-immunized calves (**Figures 5D–F**). We found a significant increase in CD4+ T-cells (**Figure 5G**; P < 0.0005) and CD8+ T-cells (**Figure 5H**; P < 0.005) in post-immunized (day-21) calves compared with pre-immunized (day-0) calves.

## 4 Discussion

Immunization and chemotherapeutic agents have been used for decades to protect against infectious organisms. Immunization is considered a safe, effective, and economic way to combat these infectious agents. The most important way to immunize is through the use of attenuated or inactivated pathogens or subunit vaccines. Scientists have prepared live attenuated and DNA-based vaccines but they have failed to cope with several infectious agents due to the failure of finding a suitable antigen to completely protect against the pathogen challenge and provide long-term immunity. The antigen not being exposed to the host’s immune system, poor immunogenicity of the antigen, and allergenicity to the host are major constraints in the development of a successful vaccine. The *in silico* approach helps us to identify polypeptides that have the potential to be exposed to the immune system of the host and induce a better immune response. Therefore, immunization has entered a new era, and the focus has been changed to chimeric or multivalent antigens, providing the possibility to link potential immunogenic molecules into a single structure, which seems to be an attractive strategy. Selecting an appropriate and potentially antigenic sequence has become far easier due to the availability of online databases, such as NCBI. Combining these sequences to design a suitable construct is carried out using various immunoinformatic tools. These tools not only link the antigenic sequences but also optimize the construct for the precise delivery into a biological system. This study describes the construction of such a vaccine with a trial vaccination against *T. annulata* in a rabbit model and cattle calves. The construct was prepared using different available immunoinformatic tools. We have made use of five different antigens representing different stages of *T. annulata* for the construction of this multi-epitope immunogenic construct.

One important criterion for potential antigen selection is the level of an antigen’s exposure opportunity to the host immune system, thus surface antigens are better candidates for this purpose (48). The preparation of the construct was carried out through *in silico* analysis using multiple immunoinformatic tools to reduce time and cost. We selected protein sequences of five different immunogenic proteins of *T. annulata*, i.e., SPAG-1, Ta9, Tams-1, Spm-2, and TaSP, for the immunogenic multi-epitope construct. These proteins are produced and secreted during the sporozoite, schizont, and piroplasm stages of the parasite. The vaccines based solely on sporozoite antigens are known to exhibit protection in theileriosis (49). A high titer of circulating antibodies is required to effectively block the circulating sporozoites but the sporozoites exist in circulation only for a few minutes (49). Therefore, complete elimination is unrealistic as even a lower number of sporozoites can evade the immune system and develop into macroschizonts after invading the leukocytes (49). Such vaccines based only on one stage of the parasite cannot confer complete protection. Therefore, we designed our construct to block the invasion of the parasite at each stage of its development in the biological system.

The purpose of a vaccine should be to potentiate an effective immune response and control overstimulatory mechanisms (19). Therefore, it is desirable to induce an immune response against both the sporozoite and schizont stages of *Theileria* for complete protection. Schizont-stage-specific CTLs can recognize peptide antigens complexed with the MHC-I molecule on the surface of infected lymphocytes. Therefore, any peptide vaccine requires T-cell receptors for the engagement of CTL for the generation of an efficient immune response (50). Cellular immunity can be transferred by CD8+ T-cells against *Theileria* parasites in vaccinated animals (48). While predicting CTL epitopes, the NetCTL server assigns a score to each peptide, which is the combination of proteasomal cleavage, the transporter associated with antigen processing transport efficiency, and HLA class I affinity. A higher score indicates a greater chance of eliciting an immune response. The NetCTL server reliability is higher than other publicly available methods for CD8+ T-cell epitope predictions (51).

The identification of B-cell epitopes is also vital for the development of immunogenic constructs (22). It is possible to precisely narrow down potential B-cell epitopes from the whole proteome of any pathogen (52) using advanced bioinformatics tools. The production of antibodies mediated by B-cells is an important factor in the elimination of exogenous parasites (53). Therefore, it is important to understand the importance of B-cell epitopes to establish persistent immunity against a certain parasite. Humoral response induced by a suitable number of B-cell epitopes can help eliminate *Theileria* infection when it comes out in the blood in the form of merozoites. The ABCPred server has better performance in the prediction of continuous (linear) B-cell epitopes of an antigen that can cross-react with an antibody, which binds to a conformational epitope (22). The higher the score, the higher the probability of being an epitope. An appropriate number of HTL epitopes results in an induced pathogen-specific immune response triggered by T-cells, and these antigens are expressed on antigen-processing cells, such as B-cells, dendritic cells, and macrophages (54).

In the first step, the amino acid sequences were retrieved from the NCBI database and the SCRATCH protein predictor was used to test their antigenic potential. The CTL, B-cell, and HTL epitopes were predicted using the NetCTL 1.2 server, ABCPred server, and IEDB server, respectively. Then a single chimeric construct was created using GPGPG, AAY, and KK linkers to link HTL, CTL, and B-cell epitopes, respectively. An adjuvant, 50S ribosomal protein L7/L12, was added to the N-terminal end to enhance the delivery system of chimeric construct in the biological system. The adjuvants linked with vaccine epitopes help in providing long-lasting immune responses (55). The 50S ribosomal protein L7/L12 increases vaccine immunogenicity (56) and is involved in the regulation of ribosomal translation but does not interact with rRNA (57). The antigenicity value on the VaxiJen v2.0 server showed that it was a probable antigen. The other parameters that need attention while designing such constructs are stability and solubility (58). The positive GRAVY score indicated the polar nature of our designed construct and suggested its hydrophobic characteristic. The ProtParam tool of ExPASy showed the stable nature of our designed construct. Furthermore, the aliphatic index showed the thermostable nature of our construct. Peptide molecules over 5–10 kDa in molecular weight are potent immunogens (59); our construct design has a hypothetical molecular weight of 48.86 kDa.

According to secondary structure analysis, 42.6% was random coil, which is a preferable structure for recognition by antibodies (59). The alpha helices protect and maintain the protein conformation during its molecular interaction (60); in our construct, α-helices were the most abundant structure (above 50%) in the construct. Tertiary structure analysis is an important step in homology modeling of proteins and it was prepared using the RaptorX server. The refinement of our three-dimensional construct was carried out using the 3D-refine web server. The refined structure was found to be reliable as Ramachandran plot analysis revealed high percentages of residues in favorable and allowed regions. The analysis revealed the construct to be of high quality and better than the RaptorX output. The construct and TLR2 were docked to analyze the interactions and stability using Cluspro. TLR2 is expressed in various immune cells and can sense a wide range of pathogen-associated molecular patterns (PAMPs) derived from various pathogens, including parasites from phylum Apicomplexa (61).

The formation of competent cells is a key technique of genetic engineering for the transformation of foreign DNA into bacterial cells. We used commercially available *E. coli* (DE3) for the transformation of our pET28a-*T. annulata* plasmid and kanamycin to check the growth of bacterial colonies on an agar plate. The growth was observed after 24 h at 37°C in positive transformed colonies. The confirmation of recombinant plasmids was carried out by extracting the plasmid containing our gene of interest, subjecting it to restriction analysis, and checking its size using agarose gel electrophoresis.

The protein size estimation in SDS-PAGE is based on the relative migration of the protein of interest to the weight markers. The migration of protein is also influenced by irregular binding of SDS, which may lead to an inaccurate apparent molecular weight. Second, the mobility of protein in SDS-PAGE is a function of molecular size rather than its weight. As the SDS-bound proteins may exist in a random coiled form due to incomplete linearization and the retention of disulfide bonds, the actual shape of the protein may differ from the ideally assumed one (62). The presence of a hairpin also affects the migration from −10% to +30% in SDS gels (63). Additionally, commercially available molecular weight markers can deviate by over 10% (62). A deviation of predicted molecular weight has also been reported in previous studies (64, 65).

The humoral immune response detected in vaccinated or infected animals is very strong, and the immunogenic proteins are believed to induce these specific antibodies with a high affinity to limit the effect of the parasite (66). In the present study, we checked the OD values to determine the level of antibodies while taking the OD value as the cutoff value of *Theileria* positive animal sera.

The main factor for the lack of effective immune response is the ability of the parasite to evade the host defense system. The evasion mechanism in *Theileria* is the abnormal activation of CD4+ and CD8+ T-cells in which macrophages act as over efficient antigen-presenting cells; this activates CD4+ and CD8+ T-cells abnormally, which helps the growth of the parasite in macrophages, allowing parasitized cells to continuously proliferate in the infected individual (67). Second, as the macroschizont stage is intracellular, it is not highly exposed to the humoral immune system and has a limited effector T-cell response. Different vaccines produced in the past against tropical theileriosis have shown variable degrees of protection. One such vaccine produced by radiating the schizont stage of *T. annulata* provided more than 67% protection under experimental conditions (68). Another vaccine with live attenuated schizonts produced through 5–15 passages in lymph node cell culture demonstrated more than 83% protection in field vaccine trials, with a limitation of protection up to 82 days only, beyond which there are no detectable antibodies; additionally, it has a short shelf life (60 days) (68). Another live attenuated vaccine was produced through 200 passages of *T. annulata* schizonts, in which the schizonts were recovered from circulation, induced more than 99% protection in the field trials (68). Rakshavac-T, a live attenuated vaccine commercially produced in India, claims to provide 95% protection but has a prolonged production time (150 *in vitro* passages) (69). Although these vaccines have been tested in different experimental and field trials, a very long production time in terms of many *in vitro* passages and maintenance of the cold chain are big challenges associated with them.

A good approach to combat *Theileria* infection in bovines is to induce an effective immune response using multiple antigens of this parasite simultaneously so that the antibodies and effector T-cells induced prepare the body ready to respond in case of an attack by a pathogenic strain under field conditions. The likelihood of such a response is greatly increased when antigenic molecules employed in the vaccine are enriched with epitopes capable of triggering B-cells and T-cells effectively. There is a big challenge in selecting potential candidates for designing such an ideal vaccine. Hence, the quality of data can significantly affect and determine the success or failure of these *in silico*-designed vaccines. Finding and selecting suitable antigens in the current study was a crucial step that strengthened the capability of the chimeric vaccine developed. The parasite possesses many different proteins, including those that interact with the host’s immune system as well as those that do not get an opportunity to have direct interaction with the immune system. Although we have selected five proteins of *T. annulata* that are known to have the ability to trigger the immune system, this may not be enough in some circumstances, such as in the case of a heavy exposure within a short period of time and in situations of immunosuppression. The current study model of testing *in silico*-designed multi-epitope recombinant protein-based vaccines paves the way for formulating highly effective vaccines against other similar pathogens in the future.

## 5 Conclusions

This study provides evidence of the successful preparation of an immunogenic construct against *T. annulata* by using immunoinformatic tools, in parallel with providing real-time evidence of its immunogenicity in a model animal (rabbit) as well as in the respective host (cattle). This design opens new avenues for multi-epitope vaccine development for other similar parasites, by providing an accurate analysis of results using *in silico* tools. Clinical trials to evaluate such vaccines will remain a challenge. Another avenue in this domain is to formulate a heterologous single vaccine against multiple challenges. This, in turn, would have a positive impact on global disease control and eradication programs.

## Data Availability

The original contributions presented in the study are included in the article/Supplementary Material. Further inquiries can be directed to the corresponding authors.
